# Biochemistry of Human Gut Microbiota: Related Diseases and Dietary Interactions

**DOI:** 10.3390/molecules31081369

**Published:** 2026-04-21

**Authors:** Sude Toydemir, Gokce Merey

**Affiliations:** 1 Nutrition and Dietetics Department, Faculty of Health Sciences, Marmara University, Istanbul 34854, Turkey; sudetoydemir@marun.edu.tr; 2 Basic Health Sciences Department, Faculty of Health Sciences, Marmara University, Istanbul 34854, Turkey

**Keywords:** microbiota, dysbiosis, SCFA, immune system

## Abstract

The human gut microbiota represents a complex and dynamic ecosystem of trillions of microorganisms that play a fundamental role in maintaining physiological homeostasis, regulating metabolism, and modulating the immune system. This narrative review explores the biochemical intricacies of the gut microbiome, focusing on the dominant phyla (Firmicutes, Bacteroidetes, Actinobacteria, Proteobacteria, Verrucomicrobia, Fusobacteria) and their specific contributions to host health. A critical emphasis is placed on the metabolic outputs of these microorganisms, such as short-chain fatty acids (SCFAs) like butyrate, which serve as vital energy sources and anti-inflammatory signaling molecules. Conversely, the review examines how dysbiosis, the disruption of microbial balance, is mechanistically linked to the pathogenesis of diverse conditions, including obesity, diabetes mellitus, inflammatory bowel disease (IBD), and gout. Furthermore, it highlights the profound impact of dietary interventions on microbial architecture, notably, how non-digestible carbohydrates promote beneficial taxa and eubiosis, while high-fat and high-sugar diets drive metabolic endotoxemia and systemic inflammation. By synthesizing current knowledge on microbial biotransformations of proteins and polyphenols, this work underscores the bidirectional relationship between nutrition and the microbiome. Ultimately, understanding these biochemical interactions is essential for developing targeted probiotic, prebiotic, and nutritional strategies to prevent and manage chronic metabolic and inflammatory disorders.

## 1. Introduction

The human microbiota is the community of bacteria, archaea and eukaryotes that colonize mostly in the gastrointestinal (GI) tract having several important functions in the human body including protection against pathogens, strengthening the immune system, controlling epithelial cell proliferation and differentiation, regulating metabolism by influencing insulin and contributing to digestion and metabolism [1]. Microorganisms convert indigestible food components and host-derived substrates into various metabolites [2]. The digestive system, particularly the colon, harbors the majority of microorganisms and contains more than 70% of the microorganisms in the body. In addition, the skin, urogenital and respiratory systems also provide a suitable environment for microbiota [3].

Bacteria are the most abundant and best-characterized members of the human gut microbiota. Recent literature suggests that the colon of a 70 kg reference adult contains approximately 3.8 × 10^13^ bacteria, a number broadly comparable to that of human cells [4]. Current molecular and culture-based evidence further indicates that the human gut harbors more than 3000 bacterial species, with individual adults typically carrying thousands of species and strains, reflecting substantial species- and strain-level diversity [5].

The balance of the intestinal microbiota is provided by the balance of the microbial composition that provides immune regulation, i.e., symbiosis [6], and the changes in the gut microbiota occur as a result of disease, antibiotic treatment, and dietary changes [7]. When the gut microbiota is disrupted, susceptibility to infection and the risk of septic shock increases. This may occur as a result of the proliferation of pathogenic gut bacteria, the priming of the immune system for a strong pro-inflammatory response, and reduced production of beneficial microbiota products [8].

Understanding the biochemical compounds synthesized by members of the gut microbiota, their mechanisms of action, and the roles of pathogenic microorganisms that are not typically part of the normal intestinal microbiota but may become influential under certain disease conditions, is essential for promoting human health. Knowledge of these microbial products and their biological effects provides a scientific basis for developing preventive and therapeutic strategies targeting microbiota-associated diseases. Accordingly, the aim of this review is to examine in detail the dominant microbial species commonly present in the gut microbiota, the bioactive compounds they produce, and the biochemical pathways through which these compounds exert their effects. In addition, selected pathogenic species that are not natural components of the healthy microbiota but may emerge during dysbiosis are discussed from a precautionary perspective. This narrative review evaluates the biochemical relationships between the gut microbiota and several prevalent diseases, highlighting potential mechanisms relevant to disease prevention and management. It aims to provide an integrated biochemical perspective on the gut microbiota, its metabolites, diet–microbiota interactions, and disease-related mechanisms. The literature was selected according to its relevance to the scope of the review, with emphasis on peer-reviewed studies addressing dominant microbial groups, microbial metabolites, mechanistic pathways, and common metabolic and inflammatory diseases.

## 2. Understanding the Composition and the Role of Gut Microbiota

Studies have shown that the gut microbiota settle into adult-like patterns, particularly during the first three years [9]. It was originally thought that the GI tract was sterile until it was colonized by microorganisms present in the environment at birth. However, the presence of microorganisms in amniotic fluid, fetal membranes, umbilical cord, placenta and meconium has now been demonstrated. It is thought that the microbiota in the fetus and newborn are derived from microbes in the mother. Accordingly, the way the baby is born and the intake of breast milk are also very important in the formation of the microbiota [10].

The colonic microbiota contains more bacteria than any other microbial community in our bodies. This large microbial community is involved in the catabolism of nutrients that cannot be broken down and absorbed by enzymes in the upper GI tract. Examples of these metabolites include SCFAs and indole derivatives, secondary bile acids, polysaccharide A [11] and several other biologically active molecules which play roles such as regulating the immune system [12], providing protection against pathogens [13], and biochemical functions of the human body. However, imbalances or alterations in the production of these metabolites may be involved in the pathogenesis of many diseases such as metabolic diseases, IBD, as well as asthma, cancer, and gynecological disorders [14].

### 2.1. The Most Abundant Species in Gut Microbiota and Their Roles

Microorganisms in our body are found on both internal and external surfaces of the human body, including the GI tract, skin, saliva, oral mucosa and conjunctiva. Bacteria far outnumber eukaryotes and archaea in the human microbiota and the gut microbiota mainly comprises bacterial phyla such as Firmicutes, Bacteroidetes, Actinobacteria, Proteobacteria, Verrucomicrobia and Fusobacteria, with Bacteroidetes and Firmicutes together accounting for approximately 90% of the microbiota in healthy adults [15]. In particular, *Lactobacillus* species from the phylum Firmicutes and *Bifidobacterium* species from the phylum Actinobacteria play crucial roles in the gut. These bacteria in the gut regulate bowel movements, produce vitamins, convert bile acids, promote mineral absorption, neutralize toxins [16] and control several other biochemical functions in the human body.

#### 2.1.1. Firmicutes

Firmicutes bacteria are found in a wide range of habitats and can be either beneficial or implicated in contexts such as the food and beverage industry and human/animal health, and although the phylum includes many genera, the most studied are *Faecalibacterium*, *Eubacterium*, *Roseburia*, *Blautia*, *Lactobacillus*, *Clostridium*, and *Ruminococcus* [17]. Members of this phylum are quite diverse in terms of morphology, physiology and Gram staining properties, which makes phenotypic characteristics insufficient for their detection or identification [18].

##### *Faecalibacterium* 

It is estimated that *Faecalibacterium*, a genus within this group, may constitute 5–6.5% of the human microbiota, occurring in lower amounts in women and the elderly and decreasing further as a result of modern lifestyle, while playing an important role in maintaining microbiota stability and exhibiting anti-inflammatory properties [19].

**Figure 1:**
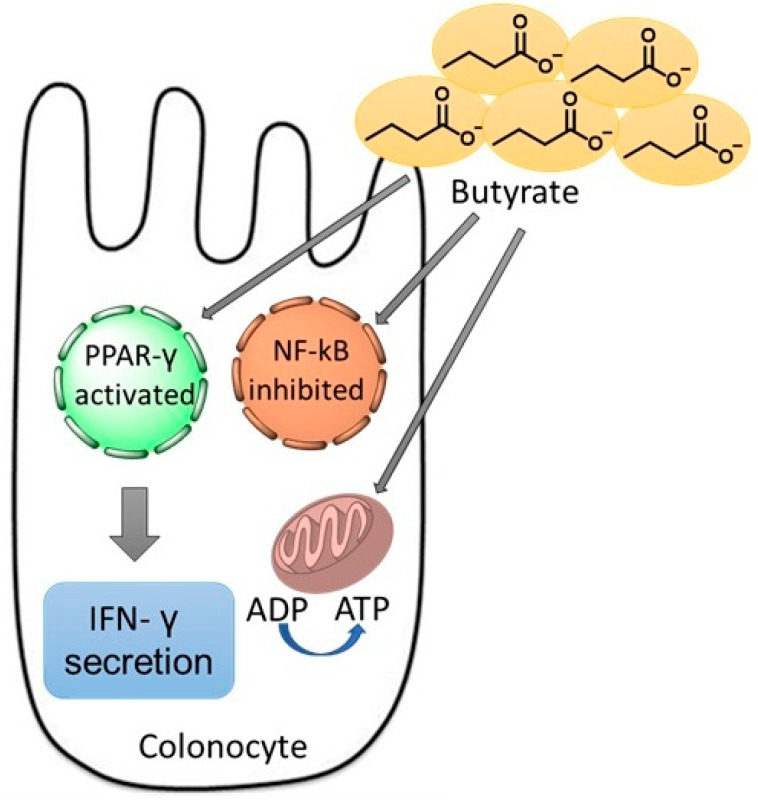
Illustration of *F. prausnitzii* protection against CRC and IBD. Butyrate supports cellular energy metabolism and modulates immune and inflammatory signaling by activating PPAR-γ and inhibiting NF-κB, thereby contributing to intestinal homeostasis.

*F. prausnitzii* is one of the most well-known members of this genus and has been consistently reported as a major butyrate producer in the gut, with butyrate serving as the primary energy source for colonocytes, offering protection against colorectal cancer (CRC) and IBD, and contributing to microbial elimination by inhibiting nuclear factor kappa B (NF-κB) activation, enhancing peroxisome proliferator-activated receptor gamma (PPAR-γ), and stimulating interferon gamma (IFN-γ) secretion [20]. Thus, *F. prausnitzii* is seen as a next generation anti-inflammatory probiotic (Figure 1) [21].

##### *Roseburia* 

Similar to *Faecalibacterium*, *Roseburia* species also produce significant amounts of butyrate from fermentable dietary carbohydrates [22]. For example, *R. intestinalis* possesses specialized enzymes and transport systems that break down β-mannans into smaller fragments, with the key enzyme RiGH26 located in the cell. These fragments are then taken into the cell by a special ABC transporter system, and within the cell, various enzymes break down these fragments into monosaccharides such as mannose and galactose. These sugars are then converted to butyrate through glycolysis [23]. Another member of this group, *R. hominis*, is a potential probiotic that reduces neuroinflammation resulting from the imbalance (dysbiosis) of the output microbiota. The benefits of *R. hominis* were highlighted through its production of propionate and butyrate which, particularly in cases of microglia loss, suppress the HDAC1 enzyme, increase histone acetylation, and reduce inflammation [24].

##### *Blautia* 

As another member of Firmicutes phylum, *Blautia* is also involved in carbohydrate fermentation and the acidification of their environment [25]. A study by Benítez-Páez group conducted in vitro experiments with *B. luti* and *B. wexlerae* strains in children to examine their anti-inflammatory effects by using interferon gamma (IFN-γ)/interleukin (IL)-4 ratios, finding that both *B. wexlerae* F15 and *B. luti* DSM 14,534 reduced the IFN-γ/IL-4 ratios, but *B. wexlerae* more effectively [26]. A one-year longitudinal study by Ozato and coworkers investigated the relationship between visceral fat area (VFA) and intestinal microbiota. As a result, *B. hansenii* and *B. producta* were found to be significantly and negatively associated with VFA accumulation and, in general, *Blautia* is found in lower amounts in individuals with some diseases compared to healthy individuals [27]. Also, *B. producta* has an anti-neuroinflammatory effect by down-regulating the COX-2/iNOS pathway [28].

##### *Lactobacillus* 

*Lactobacillus* species are the largest genus of the lactic acid bacteria (LAB) group and they metabolize carbohydrates by converting them into lactic acid. They are divided into three groups according to their metabolism: Obligate homofermentative (*L. acidophilus*, *L. salivarius*), facultative heterofermentative (*L. casei*, *L. plantarum*), and obligate heterofermentative (*L. reuteri*, *L. fermentum*). These bacteria are found in the human body and support digestion, provide protection against pathogens, and are used in fermented foods and health supplements; they can also be used in the fermentation of milk, meat, and vegetable products, and in sourdough bread-making in the industry [29]. Surface proteins (S-layer protein), extracellular polysaccharides and lipoteichoic acid found in many strains of *L. acidophilus* compete with pathogens and inhibit their binding. In addition, they have the ability to lower serum cholesterol levels, reduce the symptoms of lactose intolerance by producing the lactase enzyme necessary to digest lactose, reduce the risk of cardiovascular disease, and strengthen immunity [30]. *L. casei* also acts through mechanisms such as the production of antimicrobial substances, strengthening the epithelial barrier, competition with pathogens, and the modulation of the immune system. It has the potential to reduce the duration and symptoms of diarrhea; these effects are related to its ability to protect the intestinal microbiota during antibiotic treatment. It can remain in the intestine for a long time by binding to the mucosa by using SpaCBA pilus. It can be used preventively or competitively against vancomycin-resistant enterococci that bind with similar structures [31].

*L. plantarum* has been identified as the dominant species in various fermented vegetable products such as pickled cucumbers, sauerkraut, olives and kimchi due to its ability to grow in salty and acidic conditions and plays a protective role in food. It can also be found alone or together with other *Lactobacillus* in meats, yogurt and dairy products, and grains such as tarhana [32,33,34]. In a study, it was shown that the *L. plantarum* UM55 strain strongly inhibits the growth of aflatoxin-producing mold species by producing organic acids, especially phenyllactic acid (PLA), and suppresses toxin production [35]. However, *L. plantarum*-derived PLA can promote adipogenic activity in some cells, significantly stimulate mucosal, humoral and cellular immune responses, suppress the production of proinflammatory cytokines such as NF-κB, and reduce atherosclerotic plaque inflammation [36]. The antimicrobial and immunomodulatory effects of *L. reuteri* strains are closely related to their metabolite production profiles. One of the most important of these is reuterin which is a mixture of different forms of the compound 3-hydroxypropionaldehyde (3-HPA) produced by metabolizing glycerol via a coenzyme B12-dependent glycerol dehydratase. Although this metabolite can be produced in some other bacterial species, *L. reuteri* is unique as the only bacterium that can secrete this substance in amounts beyond its energy needs. The antimicrobial effect of reuterin depends on the spontaneous conversion of 3-HPA into a cytotoxic compound called acrolein which is effective against gastrointestinal bacterial infections of *Helicobacter pylori*, *Eshcerichia coli*, *Clostridium difficile* and *Salmonella* [37].

**Figure 2:**
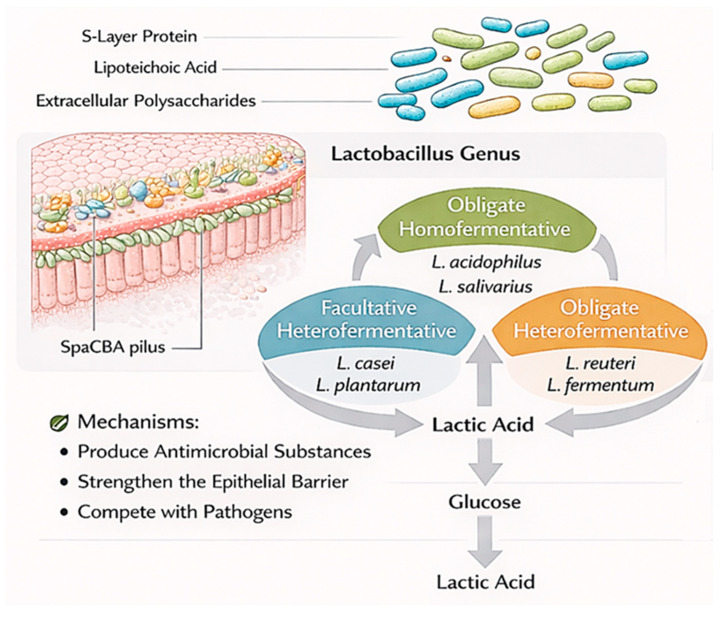
Introduction to main *Lactobacillus* species.

Main *Lactobacillus* species and their functions with mechanisms are given in Figure 2.

##### *Clostridium* 

These species are obligate anaerobic, endospore-forming, Gram-positive bacteria that include both pathogenic and commensal members of the intestinal microbiota, and many of them are capable of forming biofilms [38]. Notably, some members of this genus are of particular pathogenic significance. Firstly, *C. botulinum* is a bacterium that causes botulism, a neuroparalytic disease in humans and vertebrates. The causative agent of this bacterium is botulinum neurotoxin (BoNT), which is the most potent toxin known. BoNT, a metalloprotease, specifically cleaves soluble N-Ethylmaleimide-Sensitive Factor Binding Protein Receptor (SNARE) proteins at postsynaptic nerve terminals, thus preventing neurotransmitter release and blocking nerve conduction to effector muscles [39]. There are different types of BoNT: BoNT/A, which constitutes the most severe toxidrome; these patients usually require intubation. This disease is most commonly caused by food and has cranial involvement. Examples of its symptoms are dysarthria (common), visual disturbances, and dysphagia. In severe cases, it can rapidly progress to respiratory failure and in this case, mechanical ventilation may be required [40]. The botulinum toxin is also used for aesthetic purposes and can cause iatrogenic botulism if not used correctly [41].

*Clostridium tetani* is a noteworthy pathogenic species within the genus Clostridium, whose spores are commonly found in warm and humid environments, and which causes tetanus, a disease preventable through vaccination with inactivated tetanus toxin (toxoid). Genomic analyses indicate that this bacterium possesses numerous genes encoding amino acid transport and degradation systems as well as extracellular and intracellular peptidases and (phospho) lipases [42].

*C. difficile* is found in the human GI tract and has high molecular weight toxins called TcdA and TcdB. It has been shown that these toxins bind to epithelial cells and are taken into the cell by endocytosis, and that they glycosylate Rho proteins in the cytosol, causing the actin skeleton to deteriorate [43] and they stimulate the release of numerous proinflammatory cytokines and chemokines from epithelial cells and mucosal immune cells. This inflammatory response plays a role in determining disease severity [44]. When the balance of intestinal microorganisms is disrupted or due to wrong antibiotics use or hospital infections, the bacteria become dominant and start colonizing the large intestine and this may result diarrhea, fatal fulminant colitis, or remains asymptomatic [45].

Another bacterium of *Clostridium* genus affecting GI system is *C. perfringens* of which approximately 5% produce a toxin called *C. perfringens* enterotoxin (CPE). While most CPE positive strains are type A, it is quite common for type C and D strains to produce this enterotoxin as well. In particular, *C. perfringens* type A is seen in many food poisonings [46]. When we look at the pathogenesis of CPE, it is seen that it binds to claudin proteins and creates pores (holes) in cells which allows Ca^2+^ ions to enter the cell membrane and as a result, apoptosis is seen in low doses and necroptosis in high doses. CPE disrupts tight junction proteins in cells and increases cell membrane permeability, thereby causing damage to intestinal cells [47]. Clinically, this infection presents with diarrhea and abdominal cramps that usually resolve quickly; however, in cases of constipation or fecal obstruction, CPE may be fatal because of prolonged toxin–intestine contact, leading to systemic absorption, hyperkalemia, and death [48]. Another toxin of *C. perfringens* is the alpha toxin which is an important cause of gas gangrene and leads to hemolysis, platelet aggregation, the constriction of blood vessels, superoxide production, cytokine storm, and ultimately death. Alpha-toxin is a phospholipase C enzyme that is selectively active against two basic components found in the outer leaf of eukaryotic cell membranes, phosphatidylcholine and sphingomyelin [49,50]. Unlike other bacteria of *Clostridium* genus, *C. butyricum*, produces SCFAs such as butyrate, thus having immune modulatory effects on intestinal inflammation. In addition, this species is used as a probiotic, especially in Asian countries [51].

The phylum Firmicutes comprises numerous other bacterial species, the most prevalent ones along with their effects and mechanisms of action are presented in Table 1.

#### 2.1.2. Bacteroidetes

Bacteroidetes is a phylum of Gram-negative bacteria that colonizes diverse ecological niches and represents one of the two dominant phyla (alongside Firmicutes) in the healthy adult gut. Within this environment, its members function as commensals, mutualists, or pathobionts, with notable genera including *Bacteroides*, *Parabacteroides*, *Prevotella* and *Alistipes* [68]. In general, these species may exhibit anti-inflammatory properties and are involved in the breakdown of nutrients. They can also cause infections when the intestinal barrier is weakened and contribute to diseases by increasing the virulence of pathogens [69].

##### *Bacteroides* and *Parabacteroides*

Many studies have shown that the genus *Bacteroides* is abundant in the human intestine, having several different functions. For instance, *B. fragilis* produces the enzyme fragilysin which functions as a toxin (bft) that disrupts the intestinal epithelial barrier and contributes to disease; however, these species also show probiotic effects by modulating immune responses and maintaining gut microbial balance [70]. Some *Bacteroides* species can degrade complex sugars, as demonstrated in a study showing that the arabinogalactan of *Lycium barbarum* (LBP-3) is particularly utilized by *B. caccae* and *B. vulgatus* in pure culture experiments [71]. *B. thetaiotomicron* strains and *B. fragilis* are also capable of degrading and utilizing glycans, with a particular emphasis on mucin-type O-glycans. This property has been demonstrated to promote the synthesis of capsular polysaccharides, which are deemed to be essential for optimal colonization and persistence in the GI tract [72].

The genome of *Parabacteroides johnsonii* DSM 18315 contains a gene cluster with the *hlb5* gene which has been associated with high degradation of carboxymethylcellulose and miscanthus. In addition, this bacterium has been reported to be more abundant in patients with alopecia and may contribute to immune regulation through CD T-cell stimulation [73].

##### *Prevotella* 

The most recognized and important *Prevotella* species in the gut microbiome is *P. copri* which has been associated with inflammatory conditions such as rheumatoid arthritis, HIV infection, or ankylosing spondylitis [74]. Furthermore, *P. copri* has been shown to increase insulin resistance in mice fed a high-fat diet. However, the role of *Prevotella* species on dysbiosis and some diseases is still controversial [75].

##### *Alistipes* 

Among these species, *A. finegoldii* is reported to be protective against colitis [76] and it appears that *A. finegoldii* synthesizes membrane lipids using exogenous fatty acids found in the intestine. It has been suggested that it utilizes two different acyl-ACP synthetases (*AfAas1* and *AfAas2*) to efficiently process both medium-chain and long-chain fatty acids [77]. This mechanism may increase energy efficiency and provide a survival advantage in the gut environment. In contrast, *A. onderdonkii* has been found to be more abundant in mice with pancreatic ductal adenocarcinoma (PDAC). Although not fully understood, it is thought that it could be used as an indicator for early diagnosis of PDAC [78].

The most common bacteroidetes species, along with their effects and mechanisms of action are presented in Table 2.

#### 2.1.3. Actinobacteria

Actinobacteria are Gram-positive, branching, rod-shaped bacteria that are generally non-motile and non-spore forming. This phylum encompasses three major anaerobic families which are *Bifidobacteria*, *Propionibacteria*, and *Corynebacteria* as well as the aerobic family *Streptomyces*. Within the human intestine, *Bifidobacteria* represent the most abundant group, and their prevalence is influenced by various factors, including mode of birth [88].

##### *Bifidobacterium* 

These bacteria support other bacteria in the intestine with metabolite production and cross-feeding mechanisms and show immunomodulatory effects by increasing butyrate production. These properties vary according to their strains [89]. Studies have shown that *B. longum* enhances the body’s antioxidant activity by modulating oxidative stress and regulating reactive oxygen species (ROS) production and accumulation, thereby alleviating symptoms of diseases such as inflammatory bowel disease. At the same time, various strains of *B. longum* have been found to significantly reduce tumor necrosis factor alpha (TNF-α) levels and show anti-inflammatory properties by increasing SCFA production [90]. *B. breve* can be used to prevent some allergies. Prenatal and postnatal bifidobacteria supplementation has been shown to reduce the risk of developing eczema and atopic dermatitis in infants and significantly changed the composition and metabolic activity of the gut microbiota [91]. Among *Bifidobacterium* species, *B. bifidum* uniquely metabolizes mucin, and although mucin degradation could potentially weaken the intestinal barrier, this species may simultaneously enhance mucus layer depth by stimulating mucin production, thereby supporting epithelial barrier function, while *Bifidobacterium* spp. are also widely used as probiotics [92]. They have traditionally been used in fermented dairy products, and some strains have been recognized as Generally Recognized as Safe (GRAS) [93] due to their anti-inflammatory, anticancer, anti-virus, bone health, and fat burning effects [94].

##### *Propionibacterium* 

The genus is commonly found on the skin, although certain species have been isolated from dairy products. Notably, *P. freudenreichii* and *P. acidipropionici* from dairy differ markedly from skin-associated strains. *P. freudenreichii*, which holds GRAS status, is widely employed in fermented dairy products, particularly Swiss-style cheeses and in probiotic formulations [95]. *P. freudenreichii* has minimal nutrient requirements and synthesizes key compounds such as vitamin B12, with genetically modified strains developed to enhance the production of vitamins B2, B12, and porphyrins; it also produces cell wall-bound exopolysaccharides regulated by the GTF (glucosetransferase) gene [96].

##### *Corynebacteria* 

This group includes pathogenic bacteria, notably the toxigenic species *C. diphtheriae*, *C. ulcerans*, and *C. pseudotuberculosis*, which produce diphtheria toxin as the primary virulence factor responsible for the serious and potentially fatal human disease, diphtheria. Non-diphtheria *Corynebacteria* species are usually natural components of the human skin and mucous membrane microbiota and are not frequently found in the gut [97].

##### *Streptomyces* 

This genus is found in a variety of environments such as extreme conditions, unexplored habitats, land and marine regions, symbionts, endophytes and mangroves. *Streptomyces* produce natural products with high structural diversity such as macrolides, tetracyclines, aminoglycosides, glycopeptides, ansamycins and terpenes [98]. Although the genetic and biochemical mechanisms of gastrointestinal tolerance are not fully understood, it has been suggested that some *Streptomyces* species may have genetic rearrangements that may be resistant to acidic conditions, bile salts and digestive enzymes, suggesting their possible use as probiotics [99]. Table 3 represents the most abundant actinobacter species in gut microbiota.

#### 2.1.4. Proteobacteria

Proteobacteria represent a diverse phylum of Gram-negative bacteria that include numerous species with pathogenic potential; however, many members of this group are also present in the healthy human gut microbiota at relatively low abundance. These microorganisms are often classified as pathobionts, meaning that they can coexist with the host under normal physiological conditions but may contribute to disease development when microbial balance is disrupted. An increased relative abundance of Proteobacteria has been widely recognized as a microbial signature of dysbiosis and has been associated with inflammatory and metabolic disorders. The pathogenic potential of many Proteobacteria species is primarily related to the presence of lipopolysaccharide (LPS) in their outer membrane, which can activate host immune responses through Toll-like receptor (TLR) signaling pathways and promote systemic inflammation. Therefore, understanding both the commensal and pathogenic roles of Proteobacteria is essential for interpreting their contribution to disease mechanisms and for identifying potential microbiota-based diagnostic and therapeutic strategies [106].

This phylum is divided into 6 classes: *Alphaproteobacteria* (*Brucella* spp. and *Rickettsia* spp.), *Gammaproteobacteria* (*Escherichia* spp., *Shigella* spp., *Salmonella* spp. and *Yersinia* spp.), *Betaproteobacteria* (*Bordetella* spp. and *Neisseria* spp.), *Deltaproteobacteria*, *Epsilonproteobacteria* (*Helicobacter* spp.), and *Zetaproteobacteria* [107].

##### *Alphaproteobacteria* 

This is a widely diverse group, including both plant-associated pathogens and pathogens that cause dangerous infections in animals. In particular, the genus *Brucella* which is not a natural member of the gut microbiota in humans, is well known for causing brucellosis (Maltese fever), a severe febrile disease [108]. Human infection usually occurs through direct contact with infected cattle (*B. abortus*), sheep and goats (*B. melitensis*), pigs (*B. suis*), dogs (*B. canis*) or through consumption of unpasteurized and contaminated animal products. *B. melitensis* is the most common cause of reported cases of human brucellosis and the most severe form of the disease [109]. The BtpA/TcpB protein found in *B. abortus* and *B. melitensis* inhibits dendritic cell maturation and proinflammatory cytokine production by suppressing TLR2 and TLR4 signaling. During chronic brucellosis, the Th1 response is suppressed, and a Th2-type response dominates. Increased IL-10 production suppresses immune responses by reducing macrophage antibacterial activity, enabling *Brucella* to establish long-term infections [110].

*Rickettsia* is another genus consisting of 27 species, of which about 17 are pathogenic to humans and animals. *Rickettsia* species are not natural members of the gut microbiota; however, especially *R. typhi* and *R. rickettsii* may be detected during systemic infection rather than through true intestinal colonization. These species are divided into the typhus group (TG) and the spotted fever group (SFG). TG is transmitted by human body lice and fleas, while SFG is transmitted by mites and hard ticks [111]. *R. prowazekii* and *R. typhi* are known as the causative agents of epidemic typhus and endemic typhus diseases, respectively. As the main target cells of *Rickettsia* are endothelial cells, patients may have a characteristic haemorrhagic rash due to the damage and inflammation of local blood vessels [112]. *R. parkeri* and *R. rickettsii* rickettsiosis are considered the most common tick-borne diseases [113]. *R. conorii* is the causative agent of Mediterranean spotted fever (MSF), particularly in the Mediterranean region and surrounding countries [114]. *R. africiae* is also found in rural endemic areas such as South Africa and causes African tick bite fever (ATBF). In general, symptoms of these diseases include fever, bite marks (usually more than one), rash, headache, myalgia and regional lymphadenopathy. Laboratory findings may include elevated transaminase levels, elevated C-reactive protein and mild leukopenia [113].

##### *Gammaproteobacteria* 

*Escherichia coli* is the most studied microorganism in this class. This bacterium is both a common commensal member of the GI tract and one of the most important pathogens in humans. Some strains of this bacterium are the main cause of urinary tract infections (UTI). Such isolates have special virulence factors such as adhesins, toxins, iron acquisition systems, polysaccharide capsules and invasins that are not present in commensal and intestinal pathogenic strains [115]. Enterotoxigenic *E. coli* (ETEC) is an example of pathogenic intestinal strains, representing a major enteric pathogen responsible for tens of millions of diarrheal cases annually. ETEC initially adheres to small intestinal epithelial cells via colonization factors (CFs) and then secretes enterotoxins, primarily heat-labile (LT) and heat-stable (ST) toxins, which elevate cyclic nucleotide synthesis in the host epithelium, resulting in electrolyte and water loss and causing diarrhea [116]. Enteropathogenic *E. coli* (EPEC), another member of the pathogen group, is a non-invasive bacterium and causes diarrhea in children. EPEC reduces water absorption by disrupting the ion balance of intestinal epithelial cells, resulting in water loss and diarrhea while increasing IL-1β, TNFα and IFN-γ levels causing inflammation [117]. Adherent strains of this bacterium are named enteroaggregative *E. coli* (EAEC) and the studies showed that EAEC, like other strains, is a causative agent of diarrhea because of its intestinal colonization due to the characteristic aggregative adherence to the colonic mucosa [118]. Finally, enterohemorrhagic (Shiga toxin-producing) *E. coli* (EHEC/STEC) is a zoonotic food and waterborne pathogen that can cause serious illnesses such as bloody diarrhea (HC) and haemolytic uremic syndrome (HUS), especially in children. The main cause of the disease is damage to the kidney and other organs by Shiga toxins (Stx), especially Stx2. The pathogenicity of these bacteria is associated with toxin production as well as additional virulence factors such as the ability to adhere to host epithelial cells and locus of enterocyte effacement (LEE) [119].

*Shigella*, which is transmitted via the fecal–oral route and is passed to humans through contaminated food, water, flies, hands and inanimate surfaces is one of the oldest human-specific pathogens and is genetically very similar to *E. coli*. Four species comprise this genus: *S. dysenteriae*, *S. flexneri*, *S. boydii* and *S. sonnei* [120]. The pathogenicity mechanisms of *Shigella* are based on seven basic steps such as intestinal attachment, intracellular entry, escape from autophagy, vacuole formation and destruction, intracellular proliferation and escape from the immune system. These processes are carried out by special structures such as Type 3 Secretion System (T3SS) and virulence factors such as IcsA, IpaB, IpaC. In particular, *S. sonnei* and *S. flexneri* commonly cause mild cases of diarrhea in developed and developing countries, while *S. dysenteriae* is associated with severe dysentery [121].

*Salmonella,* which is usually transmitted through food sources such as poultry, eggs, dairy products, fresh fruits and vegetables, is generally defined as pathogenic. The most common clinical picture of infection is gastroenteritis, but bacteraemia and typhoid fever can also be seen [122]. One of the *Salmonella* species, *S. enterica* serotype *S. Typhimurium*, is an important foodborne pathogen causing a self-limiting gastroenteritis in humans characterized by fever, acute intestinal inflammation, diarrhea and the presence of neutrophils in the stool [123]. *S. enterica* possesses Type III secretion systems (T3SS) encoded by *Salmonella* pathogenicity island-1 and 2 (SPI-1 and SPI-2) during infection. The *S. Typhimurium* species produces *Salmonella* invasion proteins (Sips) and *Salmonella* outer proteins (Sops) via SPI-1. These proteins modify the actin cytoskeleton of intestinal epithelial cells, forming membrane folds and thus allowing the bacteria to enter [124].

*Yersinia* species are zoonotic agents distributed worldwide and include both pathogenic and non-pathogenic strains. The species associated with human disease include *Y. pseudotuberculosis*, *Y. enterocolitica* and *Y. pestis* [125]. *Y. pestis* is known to have evolved from *Y. pseudotuberculosis*. This bacterium is the major cause of yersiniosis infection. To evade the innate immune system, the bacterium uses mechanisms such as the inactivation of immune cells via T3SS, the non-detection of PAMPs, and the regulation of interactions with immune cells. *Y. pestis* has also been shown to induce macrophage apoptosis in lymph nodes through the T3SS-acting YopJ protein [126]. *Y. enterocolitica*, the primary causative agent of yersiniosis, is recognized as the third most common foodborne disease in the European Union. It colonizes the gut through virulence gene products, expressing the pYV plasmid to secrete factors that facilitate binding to epithelial cells, interaction with M cells, and invasion [127].

*Helicobacter pylori*, a member of epsilon-proteobacteria, is the most common cause of chronic gastritis and can lead to gastroduodenal pathologies of varying severity. These include gastric and duodenal peptic ulcer disease (PUD), gastric cancer and gastric mucosa-associated lymphoid tissue (MALT) lymphoma [128]. *H. pylori* adapts to the acidic environment to colonize the gastric mucosa, neutralizes gastric acid with the enzyme urease and binds to epithelial cells via flagella and adhesins. Toxins such as CagA and VacA disrupt the epithelial barrier, causing inflammation and damage to gastric tissue [129]. Studies have shown that *H. pylori* infection impacts autophagy in gastric tissue by downregulating the ATG16L1 gene, associated with infection severity and gastric damage, and by silencing the autophagy-related gene MAP1LC3A, which promotes uncontrolled gastric cell proliferation and increased invasiveness [130].

Several Proteobacteria species are pathogenic; although they are not all natural constituents of the gut microbiota, the most clinically relevant species are summarized in Table 4.

#### 2.1.5. Verrucomicrobia

The knowledge about the Verrucomicrobia phylum is limited due to the small number of species isolated and characterized in pure culture. Some molecular ecology techniques have shown that Verrucomicrobia is widespread in a wide range of aquatic and terrestrial habitats [151].

*Verrucomicrobium spinosum* has a protruding, wart-like cell structure and a septate cell structure shared with planctomycetes. Studies show that it has a Type III secretion system in its genome. In a study, it was observed that *V. spinosum* had pathogenic effects on invertebrate models (such as *C. elegans*), and especially increased mortality rates in worms lacking immune genes. However, the natural host of this bacterium and whether its relationship is pathogenic or symbiotic are not yet known [152].

*Akkermansia municiphila* is the most well-known bacterial species in this phylum. Its metabolites (e.g., propionic acid) are easily accessible by the host and have positive effects on the immune system through receptors such as Gpr43. It also regulates the expression of hundreds of genes in the host intestinal tissue, and most of these genes are related to immune responses. These bacteria have been detected in multiple anatomical regions of the digestive system, including the oral cavity, breast milk, pancreas, biliary tract, small and large intestines, and the appendix, where they contribute to intestinal barrier integrity and participate in syntrophic interactions. It has been observed that. *A. muciniphila* increases butyrate production when cultured with some butyrate-producing bacterial species. This suggests that the ability of *A. muciniphila* to degrade mucus contributes positively to host health by increasing the diversity of metabolites in the intestinal environment and especially butyrate levels. Additionally, during mucus degradation, the released sulfate serves as a substrate for sulfate-reducing bacteria in the colon, facilitating hydrogen sulfide production. Notably, *A. muciniphila* is proposed to possess genes involved in L-cysteine biosynthesis utilizing this hydrogen sulfide, thereby indicating a potential role in its detoxification within the intestinal environment [153,154].

#### 2.1.6. Fusobacteria

Fusobacteria are Gram-negative, non-motile, rod-shaped bacteria ranging from facultative aerobes to obligate anaerobes with fermentative metabolism, and the order comprises two families (*Fusobacteriaceae* and *Leptotrichiaceae*) encompassing nine genera. Members of Fusobacteria are common in the mucus membranes of humans and animals and can cause periodontal diseases in the oral cavity. Studies show that they are also found in the GI system, female genital area and necrotic lesions [155].

*Fusobacterium necrophorum*, an important member of the *Fusobacteriaceae* family, causes serious infections such as pharyngotonsillitis, peritonsillar abscess and Lemierre Syndrome. It can also cause severe infections such as otitis, sinusitis, mastoiditis and intracranial complications such as meningitis, abscess and sinus thrombosis in children [156]. Studies on another genus *F. nucleatum* suggest that this bacterium may be involved in the development of colorectal cancer which may spread through transient bacteremias that occur during daily activities (e.g., tooth brushing, chewing), facilitating the transport of tumors through the blood. Fap2 lectin of *F. nucleatum* helps colonization by binding to the Gal-GalNAc molecule, which is highly abundant in CRC tumors, while tumor microenvironment factors such as hypoxia and immunosuppression give the bacterium an advantage [157]. In addition, it is known that this bacterium can support cancer development by increasing the production of ROS and triggering the release of inflammatory cytokines such as IL-10 [158]. *F. varium* was also found in biopsies of patients with ulcerative colitis and induced experimental UC in mice by producing a butyrate-rich supernatant in culture. Furthermore, antibiotic treatment against this bacterium reduced the number of *F. varium*, resulting in clinical and histological improvement and supporting long-term remission [159].

*Leptotrichiaceae* species have also been associated with some serious diseases such as systemic inflammation and septic shock. *L. trevisanii*, *L. buccalis*, *L. wadei* and *L. goodfellowii* species can enter the bloodstream and cause bacteraemia in conditions such as septic shock, endocarditis and cancer. In addition, several species (especially *L. wadei*) are thought to contribute to dental caries by producing lactic acid [160].

## 3. The Most Common Microbiota Associated Diseases

Growing evidence suggests that gut microbiota dysbiosis may contribute to disease onset and progression through defined biochemical and immunological mechanisms and arise as a consequence of disease-related physiological changes. Alterations in microbial composition can disrupt intestinal barrier integrity, increase gut permeability, and promote systemic inflammation through the translocation of microbial components such as lipopolysaccharide (LPS), which activates signaling pathways and inflammatory cytokine production. Dietary factors represent one of the most important drivers of dysbiosis; diets high in saturated fats, refined carbohydrates, and ultra-processed foods have been shown to reduce microbial diversity and favor the expansion of pro-inflammatory bacterial taxa, whereas fiber-rich diets promote beneficial microbial metabolism and the production of SCFAs that support epithelial barrier function and immune regulation [161]. These findings suggest that modulation of the gut microbiota through dietary interventions, probiotics, or prebiotics may represent a promising strategy for the prevention and management of microbiota-associated diseases. Therefore, understanding the mechanistic links between dysbiosis, diet, and disease development is essential for identifying effective microbiota-targeted therapeutic approaches.

One of the primary biochemical mechanisms linking gut microbiota dysbiosis to disease involves changes in the production and signaling functions of SCFAs such as acetate, propionate, and butyrate that are produced by the fermentation of indigestible fiber and resistant starch by the gut microbiome, and can exert anti-inflammatory effects by binding to the GPR43 receptor on immune cells.

Butyrate in particular plays an important role in inhibiting colonic inflammation by suppressing NF-κB activation and reducing inflammation by inhibiting IFN-γ signaling. It also targets PPARγ to prevent inflammation in colitis [162] and the studies showed that adding more butyrate to the diet can reduce atherosclerotic lesions, increase IL-10 production and also suppress pro-inflammatory cytokines such as TNFα, IL-1β and IL-6 [163].

**Figure 3:**
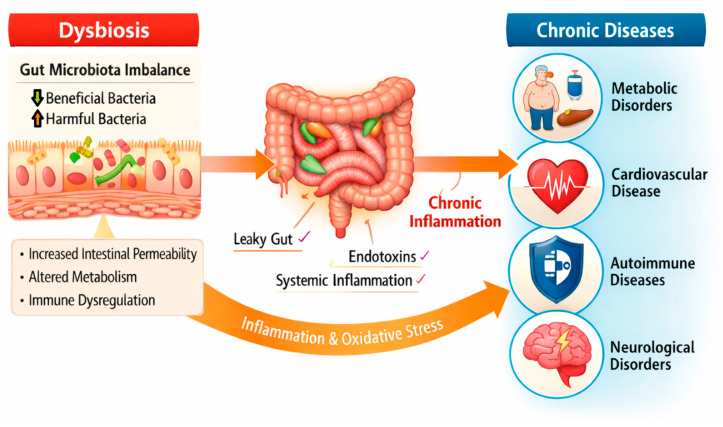
Schematic illustration of the relationship between gut dysbiosis and chronic disease. A reduction in beneficial bacteria and an increase in harmful bacteria disrupt intestinal homeostasis, leading to increased intestinal permeability, endotoxin translocation, systemic inflammation, and oxidative stress, which may contribute to the development of metabolic, cardiovascular, autoimmune, and neurological disorders.

Collectively, these observations underscore the critical role of gut microbiota derived metabolites in shaping host inflammatory and metabolic pathways. Consequently, disturbances in microbial composition and metabolite production have been increasingly recognized as key contributors to the development of microbiota associated diseases (Figure 3). In this study, we mainly focused on microbiota associated metabolic disorders. To ensure scientific clarity, it is important to distinguish between mechanisms that have been experimentally demonstrated and associations that are primarily supported by observational or epidemiological evidence. While numerous studies have identified correlations between gut microbiota alterations and disease states, causal relationships are often complex and may involve multiple host and environmental factors. Therefore, in the following sections, well-established mechanistic pathways are described where available, whereas other findings are presented as associations that may contribute to disease risk rather than definitive causes.

### 3.1. Gut Microbiota and Obesity

Obesity is a chronic complex disease defined by excessive fat deposits that can impair health and it can lead to an increased risk of type 2 diabetes and heart disease, affect bone health and reproduction, and increase the risk of certain cancers [164]. This disease is generally caused by an imbalance in nutrient intake and energy intake; However, sedentary lifestyle, gene mutations such as MC4R, leptin and POMC, and chromosomal mutations are effective in the formation of obesity [165].

Despite its simple definition, obesity is a multifactorial complex disease in which adipocytes produce and secrete a variety of biological molecules as their numbers increase. These include adipokines (leptin, adiponectin, resistin) as well as cytokines and chemokines (TNF-α, IL-6, MCP-1). These molecules have both pro-inflammatory and anti-inflammatory effects. Among the inflammatory mediators, three important factors are produced by macrophages: TNF-α, IL-6, and adiponectin. IL-6 also potently stimulates liver cells to produce and secrete C-reactive protein (CRP), a marker of inflammation [166,167]. This inflammatory state occurring in adipose tissue may contribute to local (within the adipose tissue) and systemic insulin resistance through autocrine effects on insulin signaling and metabolism. This insulin resistance can occur through several different molecular pathways. For example, these pathways are activated by cytokines, saturated fatty acids, and alarmins such as high mobility group box 1 (HMGB1) released from damaged cells. This pathway is activated in many tissues, including adipose tissue, in obesity. It suppresses insulin signaling by serine phosphorylation on insulin receptor substrate (IRS)-1 or the insulin receptor, thereby developing insulin resistance. For example, IFN-γ activates the Janus kinases (JAK1/JAK2) and signal transducers and activators of transcription (STAT)-1 pathway, and IL-6 activates the STAT3 pathway. These pathways can be activated in obesity. Both pathways activate the suppressors of cytokine signaling proteins (SOCS1 and SOCS3), suppressing the tyrosine kinase activity of the insulin receptor, disrupting the interaction with IRS proteins, and triggering the degradation of IRSs. Consequently, insulin signaling is impaired [168].

Numerous studies have demonstrated a strong association between the gut microbiota and obesity, indicating that a healthy microbial composition protects against obesity development [169,170]. Obesity related alterations in gut microbiota composition modulate host metabolism at the molecular level through alterations in microbial-derived metabolites and downstream signaling pathways [171,172]. An obesogenic microbiota enhances energy extraction and lipopolysaccharide-driven activation of TLR4–NF-κB signaling, leading to chronic low-grade inflammation, impaired insulin signaling, and metabolic endotoxemia [173,174]. In contrast, a protective microbiota is enriched in SFCA producing bacteria that activate G protein-coupled receptors (e.g., GPR41/43), inhibit NF-κB signaling, and engage PPARγ-dependent pathways, thereby reinforcing intestinal barrier integrity and promoting anti-inflammatory and metabolically favorable responses [175,176]. The molecular mechanisms distinguishing obesogenic and protective gut microbiota are summarized in Table 5.

### 3.2. Gut Microbiota and Gout Disease

Gout is the most common type of inflammatory arthritis (joint inflammation) and leads to a deterioration in quality of life. In gout, elevated blood uric acid levels (hyperuricemia) lead to the formation of monosodium urate (MSU) crystals and their accumulation in the joints. There is epidemiological evidence that the disease is currently on the rise [177].

When looking at the pathogenesis of gout, it appears that an innate immune response develops against the accumulation of MSU crystals which causes a sudden onset of painful, red, warm, and swollen joints in individuals. TLRs are activated, and MSUs in macrophages are phagocytosed, resulting in the activation of the NLRP3 inflammasome, a key mechanism of the disease. The NLRP3 inflammasome consists of NLRP3, ASC (adaptor protein), and procaspase-1. The NEK7 enzyme also plays a role in this complex that activates caspase-1, triggering the release of IL-1β and IL-18 and cell death, called pyroptosis, through gasdermin D [178,179].

There are studies suggesting that gut microbiota is affected by hyperuricemia, a common symptom of the disease. The microbiota composition is altered in individuals with hyperuricemia; for example, it is thought that bacteria with the allantoinase gene, which converts uric acid to urea, are deficient in gout patients, while bacteria with the xanthine dehydrogenase gene, which increases uric acid production, are abundant. Furthermore, due to increased inflammation, studies have shown that beneficial bacteria such as *Clostridium* and *Ruminococcus*, which produce SCFAs, are reduced in hyperuricemia mice [180]. Significant changes in the gut microbiota are observed in patients with hyperuricemia and gout. In these patients, a decrease in species belonging to the phylum Firmicutes, particularly butyrate-producing bacteria, and an increase in members of the Bacteroidetes, have been reported [181]. Concurrently, a decrease in microbial diversity, attenuation of genes involved in urate metabolism, and a significant decrease in Enterobacter species expressing uricase (urate oxidase) have been observed [182].

However, increases have been reported not only in Bacteroidetes but also in potentially harmful bacterial groups such as *Chloroflexi*, *Corynebacteriales* and *Erysipelotrichia* species in gout patients [181]. Some studies have shown that *Clostridium* species can reduce uric acid levels and therefore could be included in gut-specific probiotic formulations. Furthermore, SCFAs, particularly butyrate and acetate, are important therapeutic candidates due to their anti-inflammatory and intestinal barrier-strengthening effects. Therefore, SCFA-producing bacteria such as *Faecalibacterium prausnitzii*, *Oscillibacter*, and *Butyricicoccus*, as well as *Bifidobacterium*, may be potential probiotic targets for future interventions [183].

### 3.3. Gut Microbiota and Diabetes Mellitus

Diabetes is the medical term for diseases characterized by hyperglycemia (high blood sugar) and resulting from partial or complete insulin deficiency. Diabetes which may cause complications in many parts of the body is divided into four main types: Type 1 diabetes (T1DM), Type 2 diabetes (T2DM), gestational diabetes (GDM), and other specific types of diabetes [184]. Diabetic complications are generally classified as microvascular and macrovascular disorders, which commonly result in conditions such as retinopathy, kidney disease, nerve damage, coronary artery disease, stroke, and peripheral artery disease [185].

In type 1 diabetes, the autoimmune destruction of pancreatic β-cells leads to a deficiency in insulin secretion and simultaneously, pancreatic α-cell function is impaired, and increased glucagon secretion can be observed. In type 2 diabetes, impaired insulin secretion from pancreatic β-cells and, unlike type 1, insulin resistance (particularly in muscle, liver, and adipose tissue) are present. Obesity and nutritional status also play a significant role in the development of type 2 diabetes [186]. Although the definition of GDM is still unclear, it has been defined as “any level of hyperglycemia first recognized during pregnancy” and encompasses a wide spectrum from mildly impaired glucose tolerance (IGT) or impaired fasting glucose (IFG) detected in late pregnancy to overt diabetes (and rarely even new-onset type 1 diabetes) detected in early pregnancy (<20 weeks). This condition can increase the risk of complications during pregnancy, leading to conditions such as maternal hypertension and fetal macrosomia [187].

Although diabetes is influenced by multiple factors, studies indicate that the gut microbiota is closely associated with its pathogenesis, particularly in type 1 diabetes. Patients with T1D exhibit a less diverse and less stable gut microbiota, including a reduced abundance of *Faecalibacterium prausnitzii*, alongside impaired intestinal barrier integrity characterized by ultrastructural mucosal alterations and increased intestinal permeability observed in both human studies and animal models [188]. Several studies have reported a reduction in beneficial gut bacteria, including *Lactobacillus*, *Bifidobacterium*, *Blautia coccoides*, *Eubacterium rectale* and *Prevotella*, accompanied by an increased abundance of *Clostridium*, *Bacteroides* and *Veillonella*. In parallel, these microbial shifts are associated with the alteration of immune responses in affected patients. Furthermore, MyD88, a key adaptor protein in TLR signaling that mediates host responses to microbiota-derived molecules, has been implicated in the development of T1D, while lipopolysaccharide (LPS) from Gram-negative bacteria can impair pancreatic β-cell function by inducing proinflammatory cytokine production, with elevated LPS levels reported in patients with T1DM [189].

Type 2 diabetes is often associated with an increase in pro-inflammatory cytokines. Certain bacterial species (e.g., *R. intestinalis*, *B. fragilis*, *A. muciniphila*, *L. plantarum*, and *L. casei*) can improve glucose metabolism by increasing the anti-inflammatory cytokine IL-10. Additionally, the gut microbiota component flagellin, particularly found in the *Enterobacteriaceae* family, is increased in individuals with T2DM. Flagellin triggers a pro-inflammatory response in macrophages within the pancreatic islets via TLR5, leading to beta cell dysfunction, decreased insulin gene expression, and impaired proinsulin processing. This mechanism contributes to beta cell failure in diabetes. This microbiota alteration significantly impacts glucose homeostasis in liver, muscle, and adipose tissue. For example, *Bifidobacterium lactis* increases glycogen synthesis, *Lactobacillus gasseri* increases GLUT4 expression in muscles, and *Akkermansia muciniphila* and *Lactobacillus plantarum* regulate metabolic enzymes in the liver [190,191].

It has also been identified that specific genera including blautia, coprococcus, sporobacter, abiotrophia, parasutterella, peptostreptococcus and collinsella, generally found in relatively high abundance in T2DM patients while butyrate-producing microorganisms are significantly reduced. This group belongs to the order clostridiales, which includes the genera ruminococcus and subdoligranulum, as well as *Eubacterium rectale*, *Faecalibacterium prausnitzii*, *Roseburia intestinalis*, and *Roseburia inulinivorans* [192].

Lastly, research suggests that microbiota dysbiosis persists in the postpartum period, potentially being a predictive biomarker for T2DM. However, most studies have failed to establish a direct causal link between gut microbiota and GDM [193]. Interestingly, in a study by Hasan et al., the genus anaerotruncus was found to be more abundant in children of women with a history of GDM compared to children of mothers without GDM. This genus has been positively associated with glucose intolerance and intestinal permeability, suggesting it may play a role in the pathogenesis of diabetes [194]. In another study, while the meconium microbiota in infants of GDM mothers had lower diversity, increased firmicutes and streptococcaceae, and decreased proteobacteria, some metabolites such as riboflavin and taurine increased in the metabolome, and other metabolites such as glycerophosphocholine decreased; in the control group, microbial diversity was higher and metabolic profiles were more balanced [195].

### 3.4. Gut Microbiota and Inflammatory Bowel Diseases

Inflammatory bowel disease (IBD) which is divided into two main forms (Crohn’s disease and ulcerative colitis) is a chronic intestinal inflammation characterized by recurrences. These diseases are characterized by complex barrier dysfunction, usually evident in the terminal ileum and colon. Crohn’s disease (CD) is characterized by transmural, segmental inflammation that can affect any part of the GI tract and is often associated with complications such as abscesses, fistulas, and strictures, whereas ulcerative colitis (UC) is confined to the colon and involves inflammation limited to the mucosal layer. In these diseases, various disorders in autophagy, endoplasmic reticulum stress, and monocyte functions weaken antimicrobial defenses, and microbiota disruptions develop [196,197].

In UC, the immune system recognizes and responds to bacteria, particularly via TLRs, resulting in increased production of proinflammatory cytokines. TLR2 and TLR4 are known to be overstimulated in these patients. Bacteria adhering to and penetrating the mucosa are significantly increased in UC patients. The mucus layer is also weakened in these patients. Beneficial firmicutes species, in particular, are reduced in UC, while proteobacteria and some fusobacteria increase [198]. Furthermore, interactions between the host and fungi, viruses, and other microorganisms are also important in UC patients. An imbalance (dysbiosis) in the fungal flora is also observed in UC patients; fungal composition changes with an increase in *Candida* spp. Furthermore, the abundance of viruses such as bacteriophages in UC patients is also different compared to controls [199].

Crohn’s disease (CD), although its exact cause is unknown, is defined as a chronic and inflammatory bowel disease that affects the immune system. It is generally associated with damage to the intestinal mucosa, with periods of remission and exacerbation. It is also thought to be genetic, with mutations in the ATG16L1 and IRGM genes thought to increase the risk of CD [200]. Regarding the pathogenesis of the disease, it has been observed that the intestine overreacts to stimuli, especially in genetically predisposed individuals. Disturbances in the mucus barrier (MUC2 and FUT2 genes), an increase in TH1/TH17 cells, and an excess of inflammatory cytokines (TNF-α, IL-12, IL-23) all play a role in the disease. The interaction between the microbiota and the immune system also fuels this chronic inflammation [201]. In Crohn’s disease (CD), the gut microbiota is markedly disrupted, with reduced species richness, altered metabolite profiles, and early-onset dysbiosis characterized by decreased SCFA-producing bacteria (e.g., *Blautia*, *Clostridium IV*, *Coprococcus*, *Dorea*, and *Fusicatenibacter*), alongside increased Proteobacteria, the emergence of Fusobacterium, a reduction in the *Clostridia* cluster, and the expansion of *Enterobacteriaceae* during disease flare-ups [202,203]. Some studies have reported mixed findings, such as reduced mitochondrial protein expression in children with CD associated with a loss of beneficial butyrate-producing bacteria, alongside an increase in hydrogen sulfide–producing microbes that promote mitochondrial dysfunction and intestinal inflammation. Notably, *Atopobium parvulum* has been shown to exacerbate colitis by inducing inflammatory responses [204]. Studies have also shown that fecal microbiota can distinguish between exacerbations and remissions in CD. In one study, *F. prausnitzii* was strongly associated with disease remission, likely through its butyrate-mediated effects on intestinal health, whereas enterotoxigenic *B. fragilis* was linked to active disease and increased intestinal permeability [205].

Despite substantial progress in microbiome research, many reported relationships between microbial composition and disease remain associative, highlighting the need for longitudinal and mechanistic studies to clarify causal pathways.

## 4. Impact of Nutrients on Gut Microbiota

Specific dietary components can selectively promote or suppress particular microbial taxa by influencing the production of bioactive metabolites, affecting intestinal barrier integrity, immune regulation and systemic inflammation; therefore, they contribute to the onset or progression of metabolic and inflammatory disorders. As a result, understanding both the dietary sources of key nutrients and the overall dietary patterns in which they are consumed is essential for developing effective nutrition-based strategies for disease prevention and management. To better understand these relationships, it is important to examine how major nutrients shape gut microbial composition and metabolic activity. The following sections therefore focus on the specific effects of these nutrient groups on microbial metabolism and their potential implications for health and disease.

### 4.1. The Role of Carbohydrates in Gut Microbiota Modulation

Dietary carbohydrates are generally categorized into digestible carbohydrates (sugars and starches) and non-digestible carbohydrates (NDCs), often referred to as dietary fiber. While digestible carbohydrates are primarily absorbed in the small intestine, NDCs reach the colon intact, serving as the primary energy source for the colonic microbiota.

NDCs, including resistant starch, inulin, and fructo-oligosaccharides (FOS), function as prebiotics. These compounds selectively stimulate the growth of beneficial bacteria, particularly *Bifidobacterium* and *Lactobacillus* species. Through the process of fermentation, these species produce SCFAs, primarily acetate, propionate, and butyrate. Butyrate acts as the primary energy source for colonocytes and maintains intestinal barrier integrity while propionate and acetate enter systemic circulation, influencing metabolic health and immune signaling [206]. In addition, a recent study showed that galactooligosaccharides mediate NF-κB pathway to improve intestinal barrier function and intestinal microbiota [207]

Conversely, diets high in refined sugars (glucose, fructose, and sucrose) can induce dysbiosis. Excessive simple sugar intake is associated with a decrease in microbial diversity and an expansion of pathobionts. High-fructose diets, in particular, have been shown to increase gut permeability (“leaky gut”) and promote the growth of *Proteobacteria*, which are often associated with pro-inflammatory states [208].

Diets rich in simple sugars are consistently linked to a significant reduction in alpha diversity (the variety of species within an individual). A less diverse microbiome is inherently less resilient to environmental stressors and pathogens [209]. Furthermore, high sugar intake can stimulate the growth of mucus-degrading bacteria like *Akkermansia muciniphila* and *Bacteroides caccae* when dietary fiber is absent [210]. While *Akkermansia* is generally beneficial, in the context of a high-sugar, low-fiber diet, these bacteria may over-consume the protective mucus layer of the gut, thinning the physical barrier between the lumen and the host’s immune system [211].

Interestingly, simple sugars can actively turn off the colonization machinery of certain beneficial bacteria. Research has shown that glucose and fructose can silence the expression of the colonization factor (BT3172) in *Bacteroides thetaiotaomicron*. This prevents the bacteria from successfully adhering to and colonizing the gut, even if the host attempts to supplement their diet with fiber later on [212].

Moreover, the rapid fermentation of sugars by specific taxa can lead to spikes in metabolites that influence the gut–brain axis. Excessive sugar consumption has been shown to alter the production of neurotransmitters like serotonin (95% of which is produced in the gut) and can diminish the expression of brain derived neurotrophic factors (BDNF), potentially linking sugar-induced dysbiosis to cognitive decline and mood disorders [213,214].

### 4.2. The Role of Proteins and Amino Acids

Protein reaching the distal colon (estimated at 12–18 g per day in individuals on a standard Western diet) is primarily fermented when carbohydrate sources are depleted. Unlike carbohydrate fermentation, which yields generally beneficial SCFAs, protein fermentation produces a diverse array of metabolites, some of which are potentially toxic to the host [215,216].

When gut bacteria like *Bacteroides*, *Clostridium*, and *Fusobacterium* break down proteins, they produce several classes of metabolites including branched-chain fatty acids (BCFAs) which serve as exclusive markers of protein fermentation [217], ammonia which is a byproduct of amino acid deamination (in high concentrations, it can increase epithelial cell turnover and alter DNA synthesis) [218] and also phenolic and indolic compounds which are derived from aromatic amino acids (phenylalanine, tyrosine, and tryptophan). While some indoles are neuroprotective, others like p-cresol (derived from tyrosine) have been linked to DNA damage in colonocytes [219].

The fermentation of sulfur-containing amino acids (methionine and cysteine) by sulfate-reducing bacteria (SRB), such as *Desulfovibrio*, produces hydrogen sulfide which may have signaling roles and provide energy for colonocytes in low concentrations. However, excessive hydrogen sulfide inhibits butyrate oxidation and can damage the disulfide bonds in the mucus layer, potentially contributing to the pathogenesis of ulcerative colitis [220,221].

Amino acids also serve as precursors to signaling molecules. Tryptophan is a standout example; it is metabolized by the microbiota into indole-3-aldehyde and other indole derivatives that act as ligands for the aryl hydrocarbon receptor (AhR). This pathway is crucial for maintaining intestinal immunity and the integrity of the blood–brain barrier [213].

### 4.3. The Role of Dietary Lipids

The interaction between lipids and the microbiota occurs primarily through the modulation of microbial diversity, the production of secondary metabolites, and the maintenance of the intestinal barrier.

Chronic consumption of high-fat diets, particularly those rich in saturated fatty acids (SFAs), is consistently linked to a reduction in microbial alpha diversity [222]. This shift is characterized by an elevated Firmicutes to Bacteroidetes ratio which is a hallmark of obesity related dysbiosis that enhances energy harvest from the diet [213,222]. High fat intake also promotes the proliferation of Proteobacteria, a phylum containing many proinflammatory Gram-negative bacteria [223]. Moreover, high fat diets can facilitate the absorption of lipopolysaccharides, toxins from Gram-negative bacterial cell walls, across the intestinal barrier into the bloodstream. This process, known as metabolic endotoxemia which triggers systemic low-grade inflammation by activating TLR4-NF-κB signaling pathways [213,223]. Mechanistically, excessive SFAs impair the expression of tight junction proteins like zonulin and occludin, effectively loosening the gut barrier [224].

The chemical structure of lipids dictates their microbial impact. Saturated fatty acids found in animal fats and ultra-processed foods are associated with reduced diversity and the growth of pathobionts. They have been shown to promote the production of trimethylamine, a precursor to the cardiovascular risk biomarker TMAO, by shifting the microbiome toward species like *Desulfovibrio*. Conversely, monounsaturated and Omega-3 polyunsaturated fats (found in the Mediterranean diet) foster a more beneficial environment. These fats, especially Omega-3 polyunsaturated fats, enhance the abundance of fiber-degrading bacteria like *Prevotella* and support the growth of beneficial taxa like *Bifidobacterium*, which can suppress inflammation [225].

### 4.4. The Role of Polyphenols and Phytochemicals

Polyphenols and phytochemicals represent a unique class of dietary components and unlike macronutrients, the majority of polyphenols (roughly 90–95%) reach the colon intact, where they undergo extensive microbial biotransformation. This interaction is bidirectional; the microbiota transforms inert polyphenols into bioactive metabolites, while the polyphenols selectively modulate the microbial community [226].

Polyphenols exert prebiotic-like effects by selectively promoting the growth of beneficial bacteria while inhibiting pathobionts. Compounds such as epigallocatechin gallate (EGCG) from green tea and anthocyanins from berries have been shown to increase the abundance of *Akkermansia muciniphila* and *Bifidobacterium* species. This shift enhances the production of SCFAs, particularly butyrate, despite polyphenols not being primary fiber sources [227,228].

Most dietary polyphenols are complex molecules (e.g., glycosides or polymers) that are poorly absorbed in the small intestine. Gut microbes like *Eggerthella lenta* and *Flavonifractor plautii* possess specific enzymes to deconjugate and cleave these molecules into smaller, more bioavailable phenolic acids. For example, the transformation of ellagitannins (found in pomegranates and walnuts) results in urolithin A metabolite which is a potent inducer of mitophagy and has significant anti-aging and anti-inflammatory properties that the parent compound lacks [229].

Phytochemicals possess natural antimicrobial properties, often targeting the cell membranes of Gram-negative pathobionts. Quercetin and resveratrol have been found to inhibit the growth of *Enterobacteriaceae* and *Helicobacter pylori* without negatively impacting beneficial lactic acid bacteria. This selective “weeding” helps maintaining high microbial diversity and prevents the overgrowth of opportunistic pathogens [230].

Recent studies highlight that the phenolic metabolites can cross the blood–brain barrier. Microbial metabolites of ferulic acid and curcumin have been shown to modulate neuroinflammation by regulating microglial activation. Furthermore, these compounds activate the Nrf2 signaling pathway, which enhances the host’s endogenous antioxidant [231].

Table 6 summarizes the health effects of main dietary nutrients via gut microbiota.

Beyond the effects of individual nutrients, broader dietary patterns provide a more physiologically relevant framework for understanding diet–microbiota interactions. Fiber-rich dietary patterns, typically characterized by higher intakes of whole grains, legumes, fruits, and vegetables, generally promote saccharolytic fermentation and are associated with increased abundance in beneficial taxa, including *Bifidobacterium*, *Lactobacillus*, and other butyrate-producing bacteria, together with greater production of SCFAs, especially butyrate. These metabolites support epithelial barrier integrity, modulate immune responses, and are linked to more favorable metabolic outcomes. In contrast, Western dietary patterns, usually rich in saturated fat, refined sugars but low in fiber, are associated with reduced microbial diversity, enrichment of bile-tolerant and pro-inflammatory taxa, impaired barrier function, and greater endotoxin exposure, thereby promoting low-grade systemic inflammation and increasing metabolic disease risk [232,233].

High-protein and animal-based dietary patterns can also reshape gut microbial metabolism by increasing proteolytic fermentation in the colon, which favors the formation of metabolites such as ammonia, p-cresol, branched-chain fatty acids, and hydrogen sulfide; these compounds have been associated with impaired barrier function and mucosal stress when produced in excess. Short-term animal-based diets have additionally been shown to increase bile-tolerant microorganisms such as *Alistipes*, *Bilophila*, and *Bacteroides*, while reducing several Firmicutes involved in plant polysaccharide fermentation. By contrast, polyphenol-rich or plant-based dietary patterns tend to increase microbial diversity and are frequently associated with the enrichment of beneficial taxa such as *Bifidobacterium* and *Akkermansia*, as well as with the increased formation of SCFAs and other bioactive microbial metabolites derived from polyphenol biotransformation. Collectively, these findings indicate that dietary patterns influence host physiology not only through nutrient composition itself but also through the microbial metabolites generated in response to those diets [231,234].

## 5. Discussion

The present study reinforces the concept that the gut microbiota functions as a dynamic metabolic interface between diet and host physiology, mediating key processes in immune regulation, energy homeostasis, and systemic inflammation. Accumulating evidence indicates that dietary patterns are among the most potent modulators of microbial composition and function, thereby shaping both health and disease trajectories [235,236].

In particular, diets rich in non-digestible carbohydrates promote the expansion of beneficial taxa and enhance the production of SCFAs, such as butyrate, acetate, and propionate. These metabolites play a crucial role in maintaining intestinal barrier integrity, regulating immune responses, and suppressing inflammation through multiple signaling pathways, including G protein-coupled receptors and histone deacetylase inhibition [237]. Moreover, increased dietary fiber intake has been associated with reduced circulating markers of endotoxemia, further supporting the protective role of SCFAs against low-grade systemic inflammation [238]. Conversely, diets with high fat and refined sugar intake are strongly associated with dysbiosis, increased intestinal permeability, and metabolic endotoxemia. Mechanistically, such diets promote the expansion of Gram-negative bacteria, leading to elevated lipopolysaccharide (LPS) levels in circulation. This triggers the activation of pro-inflammatory pathways, particularly the TLR4/NF-κB axis, resulting in chronic low-grade inflammation and metabolic disturbances, including insulin resistance and obesity [239].

Importantly, these findings highlight the bidirectional nature of host–microbiota interactions. While diet shapes microbial ecology, microbiota-derived metabolites in turn influence host metabolic and immune pathways, establishing a feedback loop that can either sustain homeostasis or exacerbate disease progression. Dysbiosis has been consistently linked to a wide spectrum of chronic conditions, including inflammatory bowel disease, metabolic syndrome, and neurodegenerative disorders, emphasizing its systemic impact [240]. Another critical implication of the current findings is the growing recognition of microbiota-targeted interventions as therapeutic strategies. Approaches such as dietary modulation, prebiotics, probiotics, and emerging postbiotic therapies have shown promise in restoring microbial balance and mitigating inflammation.

Overall, the evidence supports a paradigm in which diet-induced modulation of the gut microbiota represents a central mechanism linking nutrition to metabolic and inflammatory diseases. However, despite substantial progress, considerable heterogeneity in individual responses indicates that microbiome–diet interactions are highly personalized and influenced by host genetics, environment, and baseline microbial composition.

## 6. Future Directions

Despite rapid advances in gut microbiota research, several critical gaps remain that warrant further investigation. First of all, there is a need for a deeper mechanistic understanding of how specific microbial taxa and their metabolites interact with host signaling pathways. While SCFAs and LPS have been extensively studied, emerging metabolites and their roles in host physiology remain insufficiently characterized. Future research should focus on the development of personalized nutrition strategies based on individual microbiome profiles. Longitudinal and large-scale clinical studies are required to establish causality between microbiota alterations and disease outcomes.

Inter-individual variability in microbial composition significantly influences responses to dietary interventions, suggesting that a “one-size-fits-all” approach is inadequate. Integrating multi-omics technologies including metagenomics, metabolomics, and transcriptomics, will be essential for identifying predictive biomarkers and tailoring interventions. Additionally, emerging therapeutic approaches such as fecal microbiota transplantation (FMT), engineered probiotics, and microbiota-derived postbiotics represent promising avenues for restoring eubiosis and treating chronic inflammatory conditions. However, issues related to safety, standardization, and long-term efficacy remain unresolved and must be addressed before widespread clinical implementation.

Another important direction involves exploring the gut–organ axes, including the gut–liver, gut–brain, and gut–immune axes, to better understand the systemic effects of microbiota alterations. These interconnected pathways highlight the microbiome’s role as a central regulator of whole-body homeostasis rather than a localized intestinal factor.

Finally, future research should prioritize the integration of microbiome science into public health and clinical practice. Translating current knowledge into dietary guidelines, preventative strategies, and therapeutic interventions has the potential to significantly reduce the global burden of metabolic and inflammatory diseases.

## 7. Conclusions

The biochemical synergy between the human host and the gut microbiota is a cornerstone of systemic health. As evidenced throughout this review, the gut microbiome functions as a virtual metabolic organ, converting dietary substrates into a diverse array of bioactive metabolites that influence nearly every facet of human physiology. The dominance of Firmicutes and Bacteroidetes in a healthy adult provides a resilient framework for nutrient catabolism and immune regulation. Specifically, the production of butyrate by species such as *Faecalibacterium prausnitzii* and *Roseburia* emerges as a critical defense mechanism against colorectal cancer and intestinal inflammation by reinforcing the epithelial barrier and inhibiting pro-inflammatory pathways like NF-κB.

However, the delicate balance of this ecosystem is highly susceptible to exogenous factors. The transition from symbiosis to dysbiosis serves as a primary driver for the modern epidemic of metabolic and inflammatory diseases. In conditions like obesity and Type 2 diabetes, microbial shifts lead to increased energy harvest and the translocation of lipopolysaccharides (LPS), triggering chronic low-grade inflammation and insulin resistance. Similarly, the depletion of SCFA-producing bacteria and the expansion of pathobionts within the Proteobacteria and Fusobacteria phyla are central to the exacerbation of IBD and the development of gout.

Overall, the evidence discussed in this review indicates that the gut microbiota acts as a dynamic biochemical interface between diet and host physiology. Through the production of metabolites such as SCFAs, bile acid derivatives, and other bioactive compounds, gut microorganisms influence intestinal barrier integrity, immune regulation, metabolic homeostasis, and susceptibility to disease. Accordingly, disruption of this microbial balance is closely associated with the development and progression of several chronic conditions, including obesity, diabetes, IBD, and gout. These observations further highlight that dietary patterns are among the most important determinants of microbial composition and function, shaping both the diversity of the microbiota and the metabolic outputs through which it interacts with the host.

At the same time, despite substantial progress in this field, many microbiota–disease relationships remain incompletely understood, and further mechanistic and longitudinal studies are needed to clarify causal pathways. Future research should therefore focus on integrating microbial, metabolic, immunological, and clinical data in order to identify robust biomarkers and develop more precise intervention strategies. In this context, personalized nutrition, microbiota-targeted dietary modulation, and next-generation probiotics may offer promising avenues for disease prevention and management. A deeper understanding of these host–microbe biochemical interactions may ultimately support more effective and individualized approaches to improving long-term health outcomes.

## Figures and Tables

**Table 1:** Most common Firmicutes species and their roles in gut health.

Bacteria	Effect	Mechanism of Action
*Faecalibacterium prausnitzii* [52]	Anti-inflammatory	Increased IL-10 secretion through peripheral blood monocytes, dendritic cells (DCs), and macrophages. Production of anti-inflammatory molecules such as butyrate and salicylic acid
*Faecalibacterium duncaniae* [53]	Anti-viral and anti-inflammatory	Reduction in viral load in the lungs, suppressing inflammatory cytokines; improving intestinal microbiota balance and SCFA levels
*Clostridium butyricum* [54]	Anti-inflammatory and immune support	Butyrate production by fermenting dietary fiber and undigestible carbohydrates in the intestine
*Clostridium scindens* [55]	Bile acids metabolizer	Primary bile acid metabolism via 7-dehydroxylation resulting new intermediates (12-oxoLCA, 3-oxoLCA, isoDCA, isoLCA)
*Clostridium guangxiense* and *C. neuense* [56]	Energy producer and metabolic activity	Production of H_2_, CO_2_, acetic acid and butyrate by fermentation and decomposition of organic compounds
*Eubacterium hallii* [57]	Immune support	Use of glucose, acetate and lactate for butyrate and hydrogen production, metabolization of glycerol to 3-hydroxypropionaldehyde (3-HPA, reuterin) and cobalamin (vitamin B12)
*Eubacterium limosum* [58]	Anti-inflammatory and immune support	Role in growth of intestinal epithelial cells. Reduction in the inflammatory cytokine IL-6 by the production of metabolites such as butyrate
*Blautia* spp. [59]	Estrogenic, antilipogenic, anti-inflammatory	Biotransformation of icariin and polymethoxy flavones (PMFs). 7-α-Dehydroxylation of primary bile acids, leading to the formation of lithocholic acid and deoxycholic acid.
*Roseburia intestinalis* [60]	Probiotic and anti-inflammatory	Butyrate production by fermentation of xylan and β-mannan
*Roseburia inulinivorans* [61]	Anti-inflammatory and immune support	Butyrate production by fermentation of inulin
*Ruminococcus gnavus* [62]	Anti-microbial	Adherence to mucosa, bacteriocin production; metabolic activity in carbohydrates
*Ruminococcus albus* [63]	Neuroprotection	Neuron protection from β-amyloid induced toxicity by preventing DNA damage
*Lactobacillus rhamnosus* GG [64]	Anti-microbial and anti-inflammatory	Prevention of apoptosis by Major Secreted Proteins. Stimulation of immunity via lipoteichoic acids. Anti-microbial effect via producing lactic acid
*Lactobacillus casei* [65]	Anti-microbial and anti-inflammatory	Prevention of pathogenic bacterial colonization. Increase in epithelial cell proliferation. Stimulating goblet cells for mucin production.
*Lactobacillus acidophilus* LB [66]	Bacteriostatic and immune support	Biofilm formation, intravacuolar bacteriostatic effect, production of lactic acid
*Lactobacillus gasseri* [67]	Anti-microbial, anti-inflammatory, antioxidant	Production of lactic acid, bacteriocin, and H_2_O_2_. Modulation of immune responses by inducing cytokines and antioxidant activity by scavenging reactive oxygen species

**Table 2:** The most common Bacteroidetes species and their roles in gut health.

Bacteria	Effect	Mechanism of Action
*Bacteroides fragilis* [79]	Pathogenic	Production of *B. fragilis* toxin (BFT), leading to multidrug-resistant (MDR) infections in anatomical tissues
*Bacteroides thetaiotaomicron* [80,81]	Anti-inflammatory, B12 transporter	Suppression of *C. difficile* toxin production, promotion of gut barrier regeneration
*Bacteroides vulgatus* [82]	Inflammatory	Decrease in serum valeric acid (VA) and increase in osteoclast activity, activating pro-inflammatory pathways
*Bacteroides uniformis* [83,84]	Antilipogenic	Degrading plant polysaccharides such as xyloglucan, suppressing ferroptosis in the liver and reducing free fatty acids in circulation
*Bacteroides ovatus* [85]	Therapeutic, immune support	Synthesizing the inhibitory neurotransmitter gamma-aminobutryric acid (GABA), producing SCFA, reducing tryptophan and glutamine levels
*Prevotella copri* [86]	Immune support	Producing SCFA (except propionate) by fermenting complex carbohydrates in the intestine
*Parabacteroides distasonis* [87]	Anti-inflammatory	Reduction in inflammatory cytokines with S-layer containing glycoprotein, succinic acid production
*Alistipes finegoldii* [76]	Protective against colitis	Synthesis of membrane lipids using exogenous fatty acids found in the intestine.

**Table 3:** The most common Actinobacter species and their roles in gut health.

Bacteria	Effect	Mechanism of Action
*Bifidobacterium bifidum* [100]	Anti-inflammatory	Degradation of complex carbohydrates (e.g., mucin), production of immune system modulators (e.g., TNF-α), prevention of pathogens colonizing in the intestine
*Bifidobacterium breve* [101]	Anti-allergic	Maintaining Th1/Th2 balance by suppressing the production of pro-allergic cytokines such as IL-4 and IL-5 and increasing the secretion of IFN-γ, IL-10 and TGF-β1
*Bifidobacterium adolescentis* [102]	Anti-inflammatory, antiviral	Protection of the intestinal barrier by thickening the mucus layer, inhibition of inflammation by stimulating T cell expansion and reducing NF-κB activation
*Bifidobacterium longum* [103]	Anti-inflammatory, antiviral	Enhancement of intestinal mucosal layer integrity, maintenance of T cell homeostasis, secretion of SCFAs by degradation of complex carbohydrates
*Propionibacterium freudenreichii* [104]	Anticancer	Production of SCFAs against CRC through apoptosis induction
*Collinsella aerofaciens* [105]	Inflammation	Increase in intestinal permeability, increasing the expression of inflammatory cytokines (IL-17, CXCL1, CXCL5) in some diseases

**Table 4:** The most clinically relevant species of Proteobacteria and their roles for human health.

Bacteria	Effect	Mechanism of Action
*Klebsiella pneumoniae* [131,132]	Pneumonia, urinary tract infections, liver abscesses, meningitis, bacteremia	Inducing inflammation and immune evasion through lipopolysaccharide (LPS) mediated TLR4 activation, antiphagocytic capsule formation, and enhanced survival via serum resistance and iron acquisition.
*Klebsiella oxytoca* [133]	Antibiotic associated hemorrhagic colitis (AAHC), toxin production	Inducing inflammation and mucosal injury via LPS activity and cytotoxic effects, often associated with antibiotic exposure, providing apoptosis by inhibiting DNA synthesis.
*Brucella melitensis* [109]	Acute and chronic brucellosis	Establishing chronic infection by surviving and replicating within macrophages through inhibition of phagolysosome fusion and modulation of host immune signaling.
*Brucella abortus* [110]	Brucellosis, osteoarticular disease	Persisting intracellularly by remodeling the Brucella-containing vacuole via type IV secretion system (VirB), enabling immune evasion and long-term survival.
*Rickettsia rickettsii* [134]	Rocky Mountain spotted fever	Endothelial invasion and cytosolic spread via Sca/Omp-mediated entry and actin-based motility, driving NF-κB–linked inflammation and vasculitis.
*Rickettsia typhi* [135]	Murine (endemic) typhus	Intracellular infection supported by membranolytic phospholipase activities and causing systemic febrile illness with vascular involvement.
*Commensal Escherichia coli* [136]	Normal gut microbiota	Maintaining gut homeostasis through competitive exclusion and metabolic cross-feeding without inducing inflammation.
*Escherichia coli* (ETEC) [137]	Diarrhea	Inducing secretory diarrhea via heat-labile (LT) and heat-stable (ST) enterotoxins that disrupt cAMP/cGMP signaling.
*Escherichia coli* (EPEC) [138]	Infantile diarrhea	Causing lesions via T3SS-mediated effector injection, leading to epithelial barrier dysfunction.
*Escherichia coli* (EHEC/STEC) [138]	Hemorrhagic colitis	Producing Stx to inhibit host protein synthesis and triggering systemic vascular damage.
*Shigella dysenteriae* [139]	Bacillary dysentery, severe colitis, hemolytic urinary syndrome	Invading colonic epithelial cells via type III secretion system and producing Shiga toxin, causing severe inflammation and epithelial cell death.
*Shigella flexneri* [140]	Bacillary dysentery	Inducing epithelial invasion and intracellular spread through T3SS effectors and actin-based motility, leading to intense mucosal inflammation.
*Shigella sonnei* [141]	Shigellosis	Causing inflammatory diarrhea via epithelial invasion and immune activation, resulting in milder disease compared to other species.
*Shigella boydii* [142]	Bacillary dysentery	Intestinal inflammation through epithelial invasion and cytoskeletal manipulation.
*Enterobacter cloacae* [143]	Metabolic diseases	Inhibiting the phosphorylation of AMPKα and AMPKβ, activation of SREBP-1, increasing inflammatory protein expression and activating the NF-κB signaling pathway
*Citrobacter freundii* [144]	Sporadic infections	Inducing pathogenicity in the terminal ileum and colon through characteristic aggregative adherence to HEp-2 cells
*Pseudomonas aeruginosa* [144,145]	Nosocomial infections	Inducing tissue damage and persistent infection via exotoxin secretion, biofilm formation, and LPS-driven inflammation.
*Proteus mirabilis* [146]	Inflammation, urinary tract infections	Urease-mediated urine alkalinization, enhanced motility, biofilm formation, and LPS-driven inflammation leading to urinary tract damage.
*Salmonella enterica* [147]	Salmonellozis	Invading intestinal epithelial cells via type III secretion systems, triggering LPS-mediated inflammation.
*Yersinia enterocolita* [148]	Crohn’s disease	Activating caspase-3, causing rapid destruction of autophagy, increasing the secretion of TNF-α and IL-1β.
*Yersinia pestis* [125]	Yersiniosis, pneumonic plague	Injecting Yop effector proteins via a type III secretion system to suppress phagocytosis and inflammatory signaling, enabling rapid systemic spread and septic pathology.
*Helicobacter pylori* [149]	Atrophic gastritis, peptic ulcer	Damaging gastric epithelium by cytotoxin AN (CagA) and Vacuolating cytotoxin A (VacA), reduction in stomach acids.
*Desulfovibrio* spp. [150]	IBD	Increasing H_2_S proliferation and inducing immune responses such as Th17 and Treg.

**Table 5:** Obesity related species of gut microbiota.

Bacteria	Effect on Host Metabolism	Key Mechanisms of Action
*Firmicutes*	Enhanced energy harvest and weight gain	Increased fermentation of dietary polysaccharides; higher caloric extraction, elevated SCFA availability contributing to lipogenesis
*Ruminococcus* spp.	Increased fat accumulation	Degradation of complex carbohydrates, increased monosaccharide availability and energy uptake
*Clostridium cluster XIVa* (some members)	Adiposity promotion	SCFA-mediated activation of lipogenic pathways under energy-rich conditions
*Enterobacteriaceae*	Low-grade systemic inflammation	Lipopolysaccharide (LPS) release causing metabolic endotoxemia and insulin resistance
*Escherichia coli* (pathobiont strains)	Obesity-associated inflammation	LPS-induced TLR4 activation, increased gut permeability and inflammatory signaling
*Lactobacillus*	Prolonged satiety	Lactate production as a substrate for nerve cells
*L. paracasei*	Reduced fat storage	Increased ANGPTL4 expression via PPAR α and γ. ANGPTL4 inhibits lipoprotein lipase (LPL) activity
*Akkermansia muciniphila*	Reduced body weight and improved insulin sensitivity	Strengthening of gut barrier, reduced endotoxemia, modulation of host lipid metabolism
*Bacteroidetes* (overall abundance)	Lower fat mass	Less efficient energy extraction, altered bile acid metabolism
*Faecalibacterium prausnitzii*	Anti-inflammatory, metabolic protection	Butyrate production, inhibition of NF-κB signaling, increased IL-10
*Bifidobacterium* spp.	Protection against obesity and insulin resistance	Reduced gut permeability; suppression of LPS translocation, SCFA production
*Roseburia* spp.	Improved glucose homeostasis	Butyrate-mediated enhancement of intestinal barrier and anti-inflammatory signaling

**Table 6:** Impact of dietary nutrients on gut health.

Nutrient Category	Key Microbial Shifts *	Major Metabolites and Signaling	Health Effects/Outcomes
Non-Digestible Carbohydrates (Fiber)	*Bifidobacterium* (+), *Lactobacillus* (+)	SCFAs	Enhanced barrier integrity, anti-inflammatory signaling, energy for colonocytes.
Simple Sugars	Alpha diversity (−), *Proteobacteria* (+), Mucus-degraders, e.g., *A. muciniphila,* (+)	BDNF, LPS translocation	“Leaky gut,” metabolic endotoxemia, cognitive decline, and mood disorders.
Proteins and Amino Acids	*Bacteroides* (+), *Clostridium* (+), *Fusobacterium* (+)	Ammonia, BCFAs, p-Cresol, Hydrogen Sulfide	Potential DNA damage, inhibited butyrate oxidation, risk of Ulcerative Colitis.
Tryptophan (Amino Acid)	*L. reuteri* (+), *C. sporogenes* (+)	Indole-3-aldehyde, Indole derivatives (AhR ligands)	Maintained BBB integrity, intestinal immunity, neuroprotection.
Saturated Fatty Acids (SFAs)	F/B ratio (+), Alpha diversity (−), *Desulfovibrio* (+)	LPS, Trimethylamine (TMA)	Obesity-related dysbiosis, systemic inflammation, cardiovascular risk (TMAO).
Unsaturated Fats (Omega-3)	*Prevotella* (+), *Bifidobacterium* (+)	Anti-inflammatory mediators	Suppression of inflammation, fostered eubiosis, metabolic health.
Polyphenols and Phytochemicals	*Akkermansia* (+), *Enterobacteriaceae* (−)	Urolithin A, Bioavailable phenolic acids	Mitophagy (anti-aging), Nrf2 activation (antioxidant), neuroprotection.

* (+) indicates increase, (−) indicates decrease.

## Data Availability

No new data were created or analyzed in this study. Data sharing is not applicable.
